# Pituitary stalk interruption syndrome: A rare case report and literature review

**DOI:** 10.1097/MD.0000000000023266

**Published:** 2020-12-11

**Authors:** Wei Zhang, Fang Qian, Guan Lu, Yao Wu, Rui Li, Lijuan Xia, Rui Zhao, Yi Lin, Mingyu Gu, Weiwen Chen

**Affiliations:** a Department of Endocrinology; b Department of Neurology; c Department of Pediatric Surgery; d Department of Urological Surgery; e Department of Education and Science; f Department of MRI, Qujing Affiliated Hospital of Kunming Medical University, Yunnan; g Department of Endocrinology, Shanghai First People's Hospital, Shanghai, China.

**Keywords:** adrenocortical hormone, growth hormone, pituitary stalk interruption syndrome, sex hormone, thyroid hormones

## Abstract

**Rationale:**

Pituitary stalk interruption syndrome (PSIS) is a congenital pituitary anatomical defect. It is characterized by the triad of thin or interrupted pituitary stalk, absent or ectopic posterior lobe, and hypoplastic or aplastic anterior lobe. Moreover, this condition is considered rare.

**Patient concerns:**

A 23-year-old male patient presented with a history of short stature and hypogonadism. Laboratory assessment revealed low thyroxine, cortisol, and adrenocorticotropic hormone levels, which are consistent with adrenal insufficiency without hypoglycemia. The insulin-induced hypoglycemia tolerance test finding indicated growth hormone (GH) deficiency. Moreover, magnetic resonance imaging revealed an interrupted pituitary stalk, ectopic posterior pituitary, and hypoplastic anterior pituitary. This triad of symptoms was indicative of PSIS.

**Diagnosis:**

1. PSIS;
2. hypopituitarism: secondary hypothyroidism, secondary adrenocortical dysfunction, hypogonadotropic hypogonadism, and GH deficiency;
3. sphenoid sinus cyst;
4. osteoporosis;
5. hyperinsulinism; and
6. dyslipidemia.

**Interventions:**

The patient was deficient in adrenaline, thyroxine, gonadal steroid, and GH. Thus, glucocorticoid replacement therapy was initiated, followed by euthyrox, androgen, and human chorionic gonadotropin treatment. Calcium tablets, calcitriol, and alendronate sodium were used for the management of osteoporosis. The patient was 164 cm tall, and his bone age was approximately 15 years old. However, owing to a poor economic condition, the family did not proceed with GH therapy.

**Outcomes:**

The patient did not present with adrenal or hypothyroidism crisis after receiving poly-hormonal replacement therapy. His secondary sexual characteristics began to develop. However, owing to a short treatment window period, the patient could not receive the required treatment. Hence, whether the patient would have a normal fertility function needs to be confirmed.

**Lessons:**

PSIS is a rare disease with various clinical characteristics. During the neonatal period and infancy, the signs and symptoms of PSIS are often not evident. Therefore, diagnosis is delayed. The early detection of hormone deficiency and treatment initiation can affect both the quality of life and the prognosis of patients with PSIS. Thus, the diagnosis and treatment of this disease must be improved to help patients achieve a better quality of life and to prevent reproductive health problems.

## 1 Introduction

Pituitary stalk interruption syndrome (PSIS) is a rare condition, and international databases must be established to further assess the molecular etiology of this disease. Various anterior pituitary hormone deficiencies and clinical presentations are common in PSIS. However, the function of the posterior pituitary is usually normal and intact. In the study of Zhang,^[1]^ individuals with PSIS presented with anterior pituitary hormone deficiencies, such as growth hormone (GH; 100%), gonadotropin (86.52%), corticotropin (75.28%), and thyrotropin (79.78%) deficiencies. These deficiencies were characterized by short stature, cryptorchidism, micropenis, delayed puberty, hypoglycemia, and central hypothyroidism.

In the study of Yang,^[2]^ magnetic resonance imaging (MRI) revealed the triad of absent pituitary stalk(98.3%), hypoplastic anterior pituitary (98.3%), and ectopic posterior pituitary (91.4%). Ectopic neurohypophysis was commonly observed in the infundibular recess (60.4%) and the hypothalamus (18.9%).

Herein, we assess the etiology, genetic characteristics, clinical manifestations, diagnosis, and treatment of PSIS. To help patients achieve a better quality of life and to prevent reproductive health problems, the current case study aimed to show the importance of improving the diagnosis and treatment of this disease, reducing misdiagnosis, and preventing delays in the best treatment time.

## 2 Case presentation

### 2.1 Patient history

A 23-year-old male patient sought treatment for growth delay and absence of secondary sexual characteristics. His mother found that the patient was about 10 cm shorter than his peers at the age of 6 years and that he only grew 1 cm each year. But, there was no detailed data on the height and weight of this patient during his development process. Moreover, the patient did not develop secondary sexual characteristics. At the age of 13 years, he was brought to the clinic due to failure to thrive, small stature, and bilateral cryptorchidism. Hence, GH (0.1 IU/kg/d) and thyroid replacement (levothyroxine [L-T4] 25 μg/d) therapies were initiated. However, the patient only grew 1 cm each 1 year after the treatment. The effect was unsatisfactory; thus, he spontaneously discontinued the treatment. There was no significant change in height after 18 years old, and unfortunately, his parents did not record his height or weight regularly. At the age of 23 years, he was admitted to the hospital due to the absence of secondary sexual characteristics.

His final adult height was 164 cm (growth percentile: 10^th^, from −1 to −2 SD at chronological age), which was shorter than the target height of 173 cm. However, this was not indicative of dwarfism. His upper torso measured 75 cm and the lower torso 89 cm. He presented with eunuchoid body habitus and mild kyphosis. His waist circumference was 84 cm; hip circumference, 89 cm; weight, 60 kg; and body mass index, 22.3 kg/m^2^ (weight percentile: 50^th^, 0 SD at chronological age). The patient had a child-like appearance and voice. His scrotum was not pigmented and rugated, and his stretched penile length was 3 cm. Moreover, he presented with bilateral cryptorchidism. The palpable testicular volume in the scrotum was about 2 mL, which corresponds to B1 Tanner stage 1. However, the patient did present with low hairline, and his eyes were not significantly apart. Further, hair thickening, pigmentation or thin color of the skin and mucous membrane, and pigmentation of the areola and gingiva were not observed. His vital signs and cardiovascular, digestive, nervous, motor, and ophthalmic system evaluation findings were normal.

This patient was the first child of a non-consanguineous couple. He was born full term via vaginal delivery while in complete breech presentation. His birth weight (3302 g) and length (50 cm) were normal, but he had asphyxia, APGAR score was about 6. Now he could live easy normal daily life. He graduated from technical secondary school, and engaged in printing work in a copy shop. Cognitive function (MMSE) score was 22 and daily life activity (ADL) score was 95. He had no significant medical or family history of short stature. According to his parents, his younger brother was growing normally and studies in college.

### 2.2 Endocrine evaluations

To evaluate the pituitary adrenal axis function according to the adrenocorticotropic hormone (ACTH)–cortisol (COR) rhythm, the 24-hour urinary-free cortisol test and ACTH stimulation test were performed. To assess GH secretion and adrenal cortex reserve function, the insulin-induced hypoglycemia tolerance test (ITT) was conducted. Moreover, to evaluate the function of the pituitary–thyroid axis, the thyroid-stimulating hormone (TSH), T4, T3, free T4 (FT4), and free T3 levels were measured. The gonadotropin-releasing hormone stimulation test was performed to examine the function of the hypophysial–gonadal axis. To assess testicular function, the human chorionic gonadotropin excitation test was conducted. Finally, the patient was instructed to avoid drinking water and used vasopressin to evaluate for the anti-diuretic hormone secretion function of the neurohypophysis.

### 2.3 Pituitary MRI

Pituitary MRI was performed to evaluate the hypothalamic-pituitary and pituitary stalk function. The MRI images of patients with PSIS often depict a triad of interrupted or absent or thin pituitary stalk, ectopic or absent posterior pituitary, and hypoplastic or absent anterior pituitary.^[3]^

## 3 Results

### 3.1 Pituitary adrenal axis function

The baseline COR level was low, and the ACTH–COR rhythm was abnormal. However, the ACTH level at 8AM was within the normal range (Table 1). The lesions might be in the hypothalamus or pituitary. Thus, the ACTH excitation experiment using the rapid method was performed to assess for adrenocortical secretion function. In this experiment, ACTH 250 ug was administered intravenously. Then, the patient's blood sample was collected 1 hour after injection. The results were as follows: ACTH level, >2000 pg/mL and COR level, 321.8 (normal: ≥550) nmol/L. These findings were indicative of hypoadrenocorticism.

**Table 1:** Adrenocorticotropic hormone - cortisol rhythm.

Parameters	08:00	16:00	24:00
ACTH(pg/mL)	9.2 (range: 7.2–63.3)	11.38 (range: 0–31.7)	10.65 (range: 0–15.9)
COR(nmol/L)	15.26 ↓ (range: 172–497)	16.35 ↓ (range: 71.1–286)	11.47 ↓ (range: 0–143)

ACTH = adrenocorticotropic hormone, COR = cortisol.

### 3.2 ITT

The peak GH level was <10 ug/L, which is indicative GH deficiency. The peak COR level was <500 nmol/L, which indicated decreased adrenal cortex reserve function (Table 2). The insulin-like growth factor-1 (IGF-1) level was 34.50 (range: 116–358) ng/mL. IGF-1 stimulates the division and expansion of cartilage tissues and facilitates metabolic activities, including glucose production, protein anabolism, and fat and bone metabolism. Moreover, IGF-1 levels could provide evidence for the presence of GH deficiencies.

**Table 2:** Insulin-induced hypoglycemia tolerance test values.

Parameters	08:00	08:15	08:30	08:45	09:02	09:18 Hypoglycemia	09:33	Posthypoglycemic 30 min 09:48	Posthypoglycemic 45 min 10:03	Posthypoglycemic 60 min 10:18
Fingertip blood glu (mmol/L)	5.1 (range:3.9–6.1) Novolin R 5u s.c	3.7	2.8	3.7 Novolin R 3u s.c	3.2 Novolin R 3u s.c	2.1 After exsanguinate 50% glucose 40 ml i.v	9.7	7.4	4.9	4.1
ACTH (pg/mL)	17.5 (range:7.2–63.3)	NA	NA	NA	NA	12.2	NA	11.89	13.7	15.5
COR (nmol/L)	35.03 (range:172–497)	NA	31.2	NA	NA	26.7	NA	24.71	26.37	27.3
GH (ng/ml)	<0.03 (range:0–2.47)	NA	<0.03	NA	NA	<0.03	NA	<0.03	<0.03	<0.03
Venous blood glu (mmol/L)	5.20 (range:3.9–6.1)	NA	2.90	NA	NA	2.46	NA	6.92	5.11	4.20

ACTH = adrenocorticotropic hormone, COR = cortisol,GH = growth hormone, glu = glucose, i.v = intravenous, s.c = subcutaneous, NA = not available.

### 3.3 Pituitary–thyroid function

The total thyroxine (TT4) and FT4 levels were lower than the normal range, and the TSH level was slightly higher than the normal range. The following results were indicative of secondary hypothyroidism: free T3 level, 3.37 (range: 3.1–6.5) pmol/L; FT4 level, 7.65 (range: 9–23.2) pmol/L; TT3 level, 1.77 (range: 1.1–3.4) nmol/L; TT4 level, 45.68 (range: 58.0–161.0) nmol/L; TSH level, 6.65 (range: 0.25–4) uIU/mL; reverse T3 level, 0.41 (range: 0.54–1.46) nmol/L; TG level, 5.26 (range: 0–77) ng/mL; TPOAb level, 11.14 (range: 5.0–34.0) IU/mL; TRAb level, 0.30 (range: 0.3–1.75) IU/L; and TGAb level, 26.58 (range: 10.0–115.0) IU/mL.

### 3.4 Gonadal steroids

#### 3.4.1 Routine testing of gonadal steroids

The following results were obtained: prolactin level, 13.07 (range: 2.64–13.13) ng/mL; luteinizing hormone (LH) level, <0.10 (range: 1.7–8.6) IU/mL; follicle-stimulating hormone (FSH) level, 0.64 (range: 1.5–12.5) mIU/mL; testosterone level, <0.03 (range: 1.75–7.81) ng/mL; estradiol level, <5.00 (range: 25.8–60.7) p/mL; progesterone level, <0.05 (range: 0.2–1.4) ng/mL; dehydroepiandrosterone sulfate level, 5.18 (range: 30:85–690 [at 21 years of age]).

#### 3.4.2 Gonadotropin-releasing hormone stimulation test

LH did not reach a peak and the low peak concentration of FSH was at 120 minute (Table 3). These results indicated that the patient's pituitary function was impaired and the hypophysial–gonadal axis function was affected.

**Table 3:** Gonadotropin-releasing hormone stimulating test (Gonadorelin 100ug).

Parameters	0 min	15 min	30 min	60 min	90 min	120 min
LH (mIU/mL)	<0.1 (range:1.7–8.6)	<0.1	0.12	<0.1	<0.1	0.11
FSH (mIU/mL)	0.64 (range:1.5–12.5)	0.56	0.84	0.77	0.88	0.9
T (ng/mL)	<0.03 (range:1.75–7.81)	NA	NA	NA	NA	<0.03

FSH = follicle-stimulating hormone, LH = luteinizing hormone, NA = not available, T = testosterone.

### 3.5 Antidiuretic hormone secretion function test

The patient did not present with polyphagia, polyuria, or increased nocturia, and his urine specific gravity was normal. Hence, there was no need to evaluate the antidiuretic hormone secretion function.

### 3.6 Genetic mutations

In this case, the genetic, metabolic, and endocrine disease test panel revealed 1470 gene exons and introns in the border area, and the analysis focused on the known pathogenic gene with phenotypic correlation with anterior pituitary hypofunction, growth retardation, and GH deficiency. However, we found no pathogenic/possible pathogenic/clinically significant variants that could or partly explain the patient's clinical phenotype. Negative results might be limited by genetic testing methods and the current research progress in genetic diseases or the complexity of the disease. In this case, genetic factors might not be the leading cause because genetic mutations associated with PSIS were not detected (Table 4).

**Table 4:** To analyze the genetic metabolic endocrine disease panel contained 1470 gene exons and introns in the border area.

Gene	Chromosomal location (hg19)	dbSNP ID	Describe the variation	gnomAD-EAS gene frequency	ACMG variation rating	Zygote type	Relatives verification results	
							Father	Mother
(–)	(–)	(–)	(–)	(–)	(–)	(–)	(–)	(–)

ACMG = The American College of Medical Genetics and Genomics, gnomAD-EAS = Genome Aggregation Database-East Asian, SNP = single nucleotide polymorphism, (–): negative.

### 3.7 Other test results

#### 3.7.1 Anhydrous oral glucose tolerance test (75 g) value

The following results were obtained: insulin 131.20 (range: 16.5–84.67 [at 0 minute]) pmol/L, insulin 476.9 (range: 16.5–84.67 [at 120 minute]) pmol/L. The results were indicative of hyperinsulinemia (Table 5).

**Table 5:** Anhydrous glucose OGTT (75 g) value.

Parameters	0 min	60 min	120 min
C-peptide (pmol/L)	1147 (range: 260–1730)	3750 (range: 5–6times higher than 0 min)	3206 (range: 4–5 times higher than 0 min)
Insulin (pmol/L)	131.20 ↑ (range: 16.5–84.67)	835.9 (5–10 times higher than 0 min)	476.9 ↑ (range: the same as 0 min)
Glucose (mmol/L)	4.7 (range: 3.9–6.1)	9.1 ↑ (range: 3.9–8.9)	5.6 (range: 3.9–7.8)

OGTT = oral glucose tolerance test.

#### 3.7.2 Bone metabolism based on routine examination

The following results were obtained: calcium level, 2.48 (range: 2.2–2.7) mmol/L; phosphorus level, 1.57 (range: 0.87–1.45) mmol/L; parathyroid hormone level, 34.39 (range: 15–65) pg/mL; B-CTX level, 1566.00 (range: 16–704) pg/mL; total type I collagen amino terminal lengthening peptide (P1NP) level, 285.30 (range: 15.13–58.59) ng/mL; 25(OH)D3 level, 26.22 (range: <50 nmol/L); bone alkaline phosphatase level, 40.16 (male, range: <12.3) ug/L; and calcitonin level, <2.00 (range: 0–18.5) pg/mL.

#### 3.7.3 Examination of the renin angiotensin system

The following results were obtained: angiotensin-converting enzyme level, 19.9 (range: 12–68) U/L; angiotensin level, 182.615 (erect position, normal range: 49–252) pg/mL; renin 12.791 (erect position, normal range: 4–38) pg/mL; and aldosterone level, 137.312 (erect position, normal range: 40–310) pg/mL.

#### 3.7.4 Tumor marker level

The following results were obtained: carbohydrate antigen 724 level, 53.18 (range: 0–6.9) IU/mL and neuron-specific enolase level, 18.10 (range: 0–16.3) ng/mL.

#### 3.7.5 Lipid level

The following results were obtained: total cholesterol level, 5.88 (normal level: <5.2) mmol/L; triglyceride level, 3.95 (normal level: <1.7) mmol/L; and apolipoprotein E level, 80 (range: 30–60) mg/L.

### 3.8 Imaging results

#### 3.8.1 Bone mineral density (dual energy absorptiometry: Z value)

Lumbar spine 1: -2.4, lumbar spine 2: -2.6, lumbar spine 3: -3.2, lumbar spine 4: -2.6, L1-L4: -2.8, L2-L4: -2.9, femoral neck: -0.6, Ward triangle: -0.2, trochanter major: -1.1, and total: -0.7. The results were indicative of osteoporosis.

#### 3.8.2 Anteroposterior lateral radiography of the left wrist joint and the left knee joint

The left distal ulnar and radial bone and finger joint epiphysis were not closed. The patient's bone age was about 15 years old. The left knee joint epiphysis was not closed.

#### 3.8.3 Contrast-enhanced MRI scan of the pituitary gland

Description: The thickness of the anterior pituitary was about 3 mm (normal in adults: 8 mm), and there was no high signal in the saddle region on T1-weighted imaging. In the posterior lobe of the pituitary, a high signal shadow was found in the lower margin of the hypothalamus, and enhancement was evident. The pituitary stalk is located between the pituitary upper margin and hypothalamus, and the signal intensity of MRI is equal to anterior pituitary. The enhancement is uniform. However, there was no pituitary stalk in the patient in either sagittal or coronal plane. The pituitary stalk was interrupted (normal in adults: the transverse diameter of the pituitary stalk at the optic chiasm is 3.25 ± 0.56 mm and at its pituitary insertion is 1.91 ± 0.40 mm). There was no abnormal signal shadow in and around the saddle region. Moreover, the optic chiasm was not compressed. The bilateral cavernous sinus had a clear structure. However, the posterior fossa was small, and a sphenoid sinus cyst was observed.

**Figure 1:**
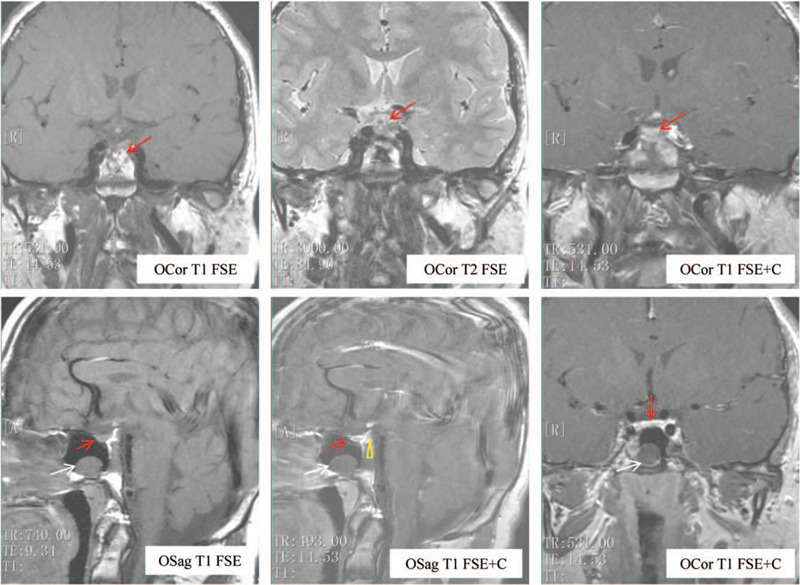
Improved findings on pituitary magnetic resonance imaging (MRI): The thickness of the anterior pituitary (red arrow) was about 3 mm, and a high signal in the saddle region was not observed on T1-weighted imaging. In the posterior lobe of the pituitary (yellow triangle), a high signal shadow was found in the lower margin of the hypothalamus, and enhancement was evident. The pituitary stalk was interrupted. There was no abnormal signal shadow in and around the saddle region. The optic chiasm was not compressed, and the bilateral cavernous sinus had a clear structure. However, the posterior fossa was small and a sphenoid sinus cyst was observed (white arrow). The MRI scan series was depicted below each image.

MRI revealed an interrupted pituitary stalk, ectopic posterior pituitary, and hypoplastic anterior pituitary. Moreover, the posterior fossa was small, and there was a sphenoid sinus cyst (Fig. 1). The images showed the typical triad of symptoms in PSIS.

#### 3.8.4 Adrenal contrast-enhanced CT scan

Bilateral adrenal contrast-enhanced CT scan revealed no abnormalities.

## 4 Diagnoses and treatment

### 4.1 Diagnoses

1) PSIS;
2) hypopituitarism: secondary hypothyroidism, secondary adrenocortical dysfunction, hypogonadotropic hypogonadism, and GH deficiency;
3) sphenoid sinus cyst;
4) osteoporosis;
5) hyperinsulinism; and
6) dyslipidemia.

### 4.2 Treatment

(1) Cortisone acetate: 25 mg per tablet, 1 tablet twice daily (medication time: 08:00 and 14:00); then, L-T4 tablets after 5 days.
(2) L-T4 (Euthyrox): 25 μg per day (medication time: 07:00, taken with an empty stomach, breakfast after 1 hour) and cortisone acetate (medication time: 08:00).
(3) Testosterone undecanoate: 40 mg soft capsule, once daily. After 1 month, 40 mg soft capsule, 1 tablet twice daily.
(4) Chorionic gonadotropin: 2000 IU, via intramuscular injection, twice a week (Monday and Thursday).
(5) Calcium carbonate and vitamin D3: 0.6 g per tablet, once daily.
(6) Alfacalcidol: 0.25 ug per tablet, once daily.
(7) Alendronate sodium: 70 mg per tablet, one tablet once a week (Tuesday) (medication time: 06:30, take with an empty stomach and ensure to stand or sit for 30 minute; take regularly with L-T4 [07:00 AM] and cortisone acetate [after breakfast at 08:00]).
(8) Follow-up examination at the endocrinology outpatient department. After 1 year, the patient began to develop secondary sexual characteristics. His scrotum was lightly pigmented and slightly rugated. His stretched penile length was 4 cm. The palpable testicular volume in the scrotum was about 3 mL. He grew 2 cm per year and he was 166 cm tall. He did not present with adrenal or hypothyroidism crisis after receiving poly-hormonal replacement therapy.

## 5 Discussion

PSIS is a congenital pituitary anatomical defect. It is characterized by the triad of thin or interrupted pituitary stalk, absent or ectopic posterior lobe, and hypoplastic or aplastic anterior lobe. Moreover, the condition is considered rare. The diagnosis is confirmed based on clinical features, endocrine evaluation results, and findings on contrast-enhanced MRI, which is the technique used to assess pituitary shape, size, and microstructure in patients with PSIS.^[4]^ In 1987, Fujisawa et al^[5]^ first described the condition, with an incidence of 0.5/100,000 births.^[6]^ The patients’ age varies. However, the typical characteristics include permanent anterior pituitary hormone deficiencies during the pediatric age, which appear gradually and progress to panhypopituitarism during adulthood.

In the current case, the patient had a child-like appearance and no secondary sexual characteristics. His baseline COR value was low, and the ACTH–COR rhythm was abnormal. However, his ACTH level at 8 AM was within the normal lower limit. The ACTH excitation experiment using the rapid method revealed hypoadrenocorticism. The peak GH level was <10 ug/L and the peak COR level was <500 nmol/L. However, the ITT revealed low GH levels and adrenal cortex reserve insufficiency. These results indicated GH deficiency and secondary adrenocortical dysfunction. The patient's TT4 and FT4 levels were lower than the normal range, and the TSH level was slightly higher than the normal range. The results were indicative of secondary hypothyroidism. The gonadotropin-releasing hormone stimulation test revealed that LH did not reach a peak and the low peak concentration of FSH was at 120 minute. The pituitary function of the patient was impaired, and the hypophysial–gonadal axis function was affected. These results indicated hypogonadotropic hypogonadism. The improved MRI images revealed the typical triad of symptoms in PSIS. Based on the clinical features and endocrine evaluation and MRI findings, the patient was diagnosed with PSIS.

The etiology of PSIS is not known. The proposed pathophysiological mechanism of PSIS is mutation in the genes (PIT1, PROP1, LHX3/LHX4, PROKR2, OTX2, TGIF, HESX1,^[7]^ ROBO1,^[8]^ and GPR161^[9]^) involved in the development of the anterior pituitary. Undescended testes and micropenis might be associated with genetic mutations in the HESX1, LHX4, and SOX3 genes.^[10,11]^ Moreover, breech presentation at the time of delivery and perinatal asphyxia might damage the pituitary stalk.^[12]^ In this case, there was no pathogenic/possible pathogenic/clinically significant variants that could or partly explain the patient's clinical phenotype. The only significant history was breech birth. This information partly provided reference for the etiology of the disease. Hence, the etiology and prognosis of PSIS should be further assessed.

After a year of treatment, at the age of 13 years, the patient discontinued taking his GH medications because the effect was unsatisfactory. His final adult height was 164 cm, which was shorter than the target height of 173 cm. However, this was not indicative of dwarfism. Various hormones, such as GH, thyroid hormone, and gonadal steroids, can affect growth. However, GH plays an essential role during the growth phase.^[13]^ Normal growth without GH can be attributed to hyperinsulinemia, hyperprolactinemia, elevated leptin levels, and GH variants.^[14]^ Elevated insulin and/or prolactin concentrations can normalize IGF-1 levels and can change the distribution of circulating IGF-1. In addition, elevated leptin levels secreted from increased fat tissues caused by obesity may be a mechanism involved in the maintenance of normal growth velocity.^[15]^ In this case, the patient was not obese. In fact, he presented with subnormal GH, thyroid hormone, gonadal steroid, and normal prolactin levels. However, he experienced hyperinsulinism and dyslipidemia. Normal growth could be explained by hyperinsulinemia and elevated leptin levels, which might be caused by dyslipidemia.

Adrenal corticosteroids can help retain sodium in the body, excrete potassium, and manage stress. As a response to stress, the hypothalamus releases corticotropin-releasing hormone, which stimulates the anterior pituitary to release ACTH. Then, the adrenal cortex upregulates the production of COR, which is the primary hormone that responds to human stress. A low COR level causes decreased intravascular volume, cardiac output, and renal perfusion. The clinical feature of acute adrenal crisis is circulatory collapse.^[16]^ Patients may present with symptoms, such as nausea, vomiting, fever, and mild chest and/or abdominal pain along with low blood pressure.^[17]^ Thyroid hormones have complex effects on the metabolism of sugar, fat and protein, thereby improving nerve excitability and promoting growth and development. Hypothyroidism is caused by a decreased production of thyroid hormones due to several causes. Myxedema coma is the most severe, life-threatening effect of severe hypothyroidism. Commonly, there is a history of discontinuation of thyroid hormone replacement therapy. In the pre-comatose state, the typical clinical findings include hypothermia, decreased mentation, and generalized edema. Based on the patient's history, he had hypopituitarism, secondary hypothyroidism, and secondary adrenocortical dysfunction. Although the thyroid hormone replacement therapy was discontinued, the patient never experienced adrenal or hypothyroidism crisis. This may be attributed to the low levels of ACTH, COR, and thyroid hormones, which could maintain the patient's metabolism in a non-stress state.

Treatment was based on substituting hormones with lifelong poly-hormonal replacement therapy. The early detection of hormone deficiency and treatment initiation affect both the quality of life and the prognosis of patients with PSIS.^[18–20]^ The patient was deficient in adrenaline, thyroxine, gonadal steroids, and GH. Glucocorticoid replacement was the initial treatment, followed by Euthyrox, androgen, and human chorionic gonadotropin. Calcium tablets, calcitriol, and alendronate sodium were used for the management of osteoporosis. The patient was 164 cm tall and his bone age was about 15 years old. However, owing to a poor economic condition, the family did not proceed with GH therapy.

## 6 Conclusions

PSIS is a rare condition and it has complex clinical syndromes. The etiology remains unknown. However, this disease might be attributed to gene–environment interactions. GH plays an essential role during the growth phase. However, other various hormones can also affect growth. The early diagnosis of PSIS is based on MRI findings. Then, prompt hormonal replacement therapies are provided. The diagnosis of this patient was delayed, and the prognosis was difficult to predict. Hence, the understanding of PSIS in clinical practice, particularly early diagnosis and timely treatment among newborn or children, should be improved. Therefore, adverse effects on long-term growth and development are prevented.

## Acknowledgment

We thank the patient and his family for their participation in this study.

## Author contributions

**Conceptualization:** Ming-yu Gu, Wei-wen Chen.

**Data curation:** Rui Li, Lijuan Xia, Rui Zhao.

**Formal analysis:** Guan Lu, Yao Wu.

**Funding acquisition:** Wei Zhang, Wei-wen Chen.

**Investigation:** Fang Qian, Guan Lu.

**Supervision:** Yi Lin, Ming-yu Gu, Wei-wen Chen.

**Visualization:** Wei Zhang, Fang Qian.

**Writing – original draft:** Wei Zhang, Fang Qian.

**Writing – review & editing:** Yi Lin, Ming-yu Gu, Wei-wen Chen.
